# The Interaction between Ambient PM_10_ and NO_2_ on Mortality in Guangzhou, China

**DOI:** 10.3390/ijerph14111381

**Published:** 2017-11-13

**Authors:** Yuzhou Gu, Hualiang Lin, Tao Liu, Jianpeng Xiao, Weilin Zeng, Zhihao Li, Xiaojuan Lv, Wenjun Ma

**Affiliations:** 1 Guangzhou Center for Disease Control and Prevention, Guangzhou 510440, China; yuchoukoo@foxmail.com; 2 Guangdong Provincial Institute of Public Health, Guangdong Provincial Center for Disease Control and Prevention, Guangzhou 511430, China; hualianglin@gmail.com (H.L.); gztt_2002@163.com (T.L.); jpengx@163.com (J.X.); letitiazeng@foxmail.com (W.Z.); zhihaoli1990@163.com (Z.L.); 2012lvxiaojuan@sina.cn (X.L.)

**Keywords:** air pollution, interaction, mortality, Guangzhou, generalized additive model

## Abstract

Air pollution is now a significant environmental issue in China. To better understand the health impacts of ambient air pollution, this study investigated the potential interaction between PM_10_ and NO_2_ on mortality in Guangzhou, China. Time series data of daily non-accidental mortality and concentrations of PM_10_ and NO_2_ from 2006 to 2010 were collected. Based on generalized additive model, we developed two models (bivariate model and stratified model) to explore the interaction both qualitatively and quantitatively. At lag of 0–2 days, greater interactive effects between PM_10_ and NO_2_ were presented in the graphs. Positive modified effects were also found between the two pollutants on total non-accidental death and cardiovascular death. When the NO_2_ concentration was at a high level (>76.14 μg/m^3^), PM_10_ showed the greatest excess relative risk percentage (ERR%) for total non-accidental mortality (0.46, 95% CI: 0.13–0.79) and cardiovascular disease mortality (0.61, 95% CI: 0.06–1.16) for each 10 μg/m^3^ increase. During the period of high PM_10_ concentration (>89.82 μg/m^3^), NO_2_ demonstrated its strongest effect for total non-accidental mortality (ERR%: 0.92, 95% CI: 0.42–1.42) and cardiovascular disease mortality (ERR%: 1.20, 95% CI: 0.38–2.03). Our results suggest a positive interaction between PM_10_ and NO_2_ on non-accidental mortality in Guangzhou.

## 1. Introduction

Ambient air pollution has long been a prominent environmental health issue. Several major air pollutants, including particulate matter with aerodynamic diameter less than 10 μm (PM_10_) and nitrogen dioxide (NO_2_), have been found to cause adverse effects on both morbidity and mortality in research worldwide [1,2,3]. However, previous studies were mainly focused on the health effects of various individual air pollutants. Actually, air pollution usually exists in the form of a complex mixture [4], to which human bodies are exposed in the ambient environment. Therefore, it is inappropriate to simply evaluate the health risk of a single pollutant. Moreover, a number of laboratory studies have found that the overall health effects of air pollutants cannot be simply calculated through the direct addition of each individual effect [4], suggesting potential interactions among air pollutants. Therefore, it is important to understand these possible interactions, so as to assess the overall health risk of air pollution.

Both PM_10_ and NO_2_ are the major air pollutants which originate from coal burning or vehicle emissions, etc. Only a few epidemiological studies have examined the interactive effect between PM_10_ and NO_2_ on mortality, and these have come to inconsistent results. For instance, some studies have suggested that the interaction between PM_10_ and NO_2_ would mutually amplify the risk of mortality [5,6,7,8], while no sufficient evidence of the interaction is found in other studies [9,10,11]. Such inconsistency may be potentially ascribed to the differences in study location and population characteristics. Therefore, it is necessary to further explore this research question in different areas.

Air pollution has recently aroused widespread concern in China, but few studies in China have focused on the interactions among air pollutants [9,12,13]. Guangzhou, the core city of Pearl River Delta (one of the major economic centers in China) in southern China, is now suffering from similar air pollution problems as other fast-growing cities in the country. Aiming to accurately assess the health impacts of ambient air pollution in Guangzhou, the current study attempted to investigate the interactive effect between PM_10_ and NO_2_ on mortality using data collected from 2006 to 2010 in Guangzhou.

## 2. Materials and Methods

### 2.1. Data Sources

#### 2.1.1. Setting

Guangzhou, one of the first-tier cities in China, is also the capital city of Guangdong Province, the most populous province. It belongs to the subtropical monsoon climate region and the average annual temperature and rainfall are 22 °C and 1800 mm, respectively. According to the Sixth Population Census in 2010, the total area of Guangzhou City reached 7434 km^2^, which was divided into 11 districts, with the population of 12.7 million [14]. In the present study, two central districts (Yuexiu and Liwan) were selected based on the following reasons: firstly, people living in these two districts are mostly permanent residents and share relatively homogeneous characteristics. Secondly, mortality data from these two districts are also of high quality [15,16]. According to statistics, there were 1.9 million residents living in the study area at the end of 2010 [17].

#### 2.1.2. Mortality Data

We obtained the daily mortality data from the Guangdong Provincial Center for Diseases Control and Prevention for the period from 1 January 2006 to 31 December 2010. According to the International Classification of Diseases, Tenth Revision (ICD-10) [18], accidental deaths were excluded and mortality was defined as total non-accidental death (ICD-10: A00–R99). Several studies have found that the interaction between PM_10_ and NO_2_ could cause adverse effects on both the cardiovascular system and the cerebrovascular system [13,19]. Consequently, the mortality was further divided into cardiovascular death (ICD-10: I00–I51, I70–I99) and cerebrovascular death (ICD-10: I60–I69).

#### 2.1.3. Air Pollution and Meteorological Data

**Figure 1:**
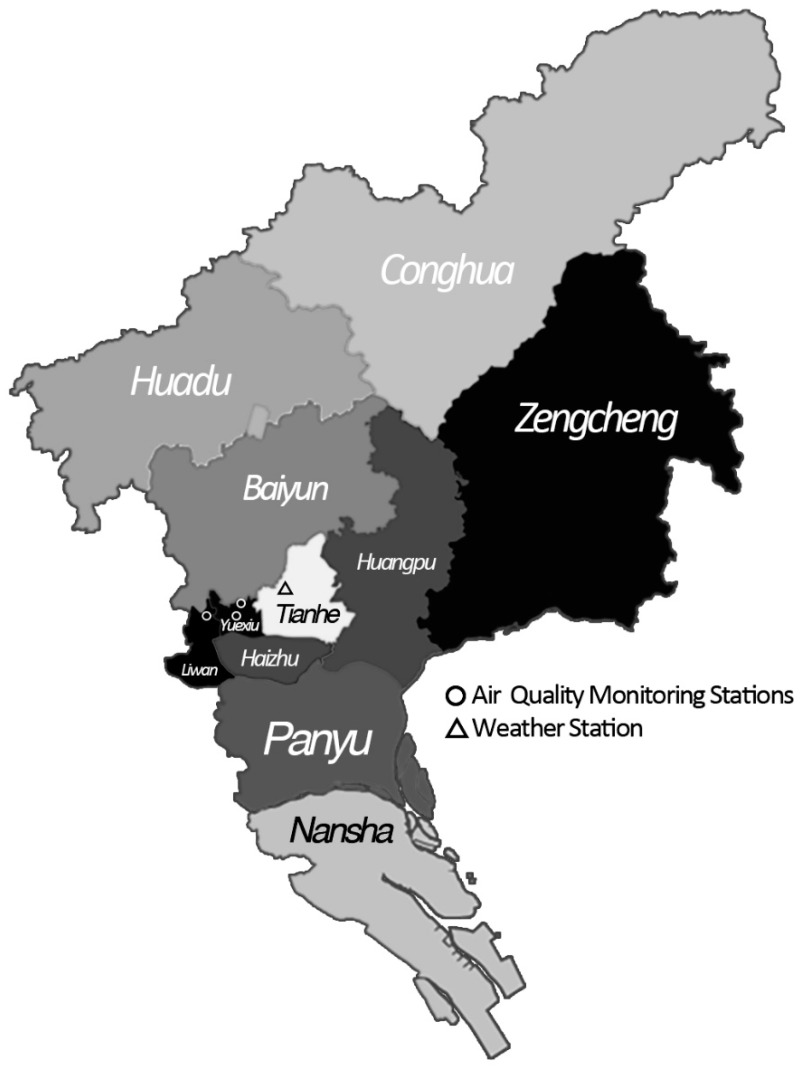
Administrative division of Guangzhou City and the locations of the monitoring stations. This figure illustrates the locations of the study area (Yuexiu District and Liwan District) and the monitoring stations which measured the data of air pollutants and meteorological factors used in the present study.

Air pollution data during the study period were collected from the Guangzhou Environmental Monitoring Center. Daily concentrations of PM_10_ and NO_2_ are arithmetic means of the 24-h average concentrations, which were measured by three monitoring stations covering the study area (Figure 1).

Meteorological data during the same period were collected from the Guangdong Provincial Climate Center, including daily mean temperature and relative humidity. Data were measured by the National Basic Weather Station of Guangzhou, the location of which is shown in Figure 1.

### 2.2. Statistical Analysis

#### 2.2.1. Core Model Development

The effect of air pollution on mortality was assessed using the time series approach. At first, the core model was built based on generalized additive model (GAM) with Poisson link without air pollution data [15,16]. The long-term trend and meteorological factors were controlled by smoothing spline function, and days of the week (DOW) was adjusted through the dummy variable in the core model:

(1) $$\log\left[E\left(Y_{t}\right)\right]=\alpha +s\left({\mathrm{Time}}_{t}\right)+s\left({\mathrm{Temp}}_{t}\right)+s\left({\mathrm{RH}}_{t}\right)+{\mathrm{DOW}}_{t}$$

where *α* is the intercept; *E*(*Y_t_*) stand for the expected number of the specific death cause on day *t*; *s*(*Time_t_*), *s*(*Temp_t_*) and *s*(*RH_t_*) indicate the smoothing variables, which represent the long-term trend, mean temperature and relative humidity on day *t*, respectively. The initial value of degree of freedom (df) for each smoothing variable was selected based on effective degree of freedom (edf) which derived from the “mgcv” package in R 3.3.1 [20] and the non-cited previous studies. Moreover, each df was further adjusted by examining the partial autocorrelation function (PACF) plot of the residuals of the core model. Df rendering the most balanced PACF plot between the upper and the lower limits of confidence interval would be chosen. Notably, the smoothing patterns of variables might differ in fitting with different death causes, thus df for each model was considered specifically. Dfs of 6 per year were selected for the long-term trend in all other models, except for that of the cerebrovascular death model (4 per year). Meanwhile, dfs of 3 and 2 were chosen for the daily mean temperature and the relative humidity, respectively.

#### 2.2.2. Single Pollutant Analysis

Secondly, the single pollutant models of PM_10_ and NO_2_ were developed through adding the daily concentrations into the core models, respectively. Besides, the lag effects of both single-day and multiple-day moving average for up to 3 days before the deaths (lag 0, 1, 2, 3, 0–1, 0–2, 0–3) were also taken into consideration. The single pollutant model is shown as follows:

(2) $$\log\left[E\left(Y_{t}\right)\right]=\alpha +\beta *{\mathrm{AP}}_{l}+\mathrm{COVs}$$

where *AP_l_* denotes the concentration of air pollutant (PM_10_ or NO_2_) at the time lag of *l* days, and *COVs* represents all the confounders in the core model (1). Excess relative risk percentage (ERR%) of mortality was calculated for every 10 μg/m^3^ increases in the concentrations of single PM_10_ and NO_2_ based on the relative coefficient (*β*). The strength of ERR% would be taken into account when selecting the time lag for further interaction analysis.

#### 2.2.3. Interaction Analysis

Two interaction models were built in the current study to examine the potential interaction between PM_10_, NO_2_ on mortality. We simultaneously fit the concentrations of PM_10_ and NO_2_ with one smoothing spline in the core model (1) to develop the bivariate model at the first step:

(3) $$\log\left[E\left(Y_{t}\right)\right]=\alpha +{\mathrm{te}(\mathrm{PM}}_{10_{l}},\;{\mathrm{NO}}_{2_{l}})+\mathrm{COVs}$$

where *te*(*PM*_10_*l*__, *NO*_2_*l*__) is the smoothing variable, which represents the joint of PM_10_ and NO_2_ at the lag days selected in the last step. We attempted to visually explore the interactive pattern of these two pollutants on mortality in the joint effect graph, which was generated by the bivariate model. Dfs for the smoothing variables were 3 for the total non-accidental death model, and 2 for both the cardiovascular death model and the cerebrovascular death model.

Subsequently, the interaction was quantitatively investigated using the stratified model:

(4) $$\log\left[E\left(Y_{t}\right)\right]=\alpha +\beta *{(\mathrm{PM}}_{10_{l}}:\;{\mathrm{NO}}_{2}\;\mathrm{level})+\mathrm{COVs}$$

(5) $$\log\left[E\left(Y_{t}\right)\right]=\alpha +\beta *{(\mathrm{NO}}_{2_{l}}:\;{\mathrm{PM}}_{10}\mathrm{level})+\mathrm{COVs}$$

To be specific, we categorized the concentrations of PM_10_ and NO_2_ into three levels (low, medium and high) with the cutoffs at 25th and 75th percentiles, respectively. Equation (4) was built to assess the ERRs (%) for each 10 μg/m^3^ increase in concentration of PM_10_ across different levels of NO_2_, while Equation (5) was used to examine the modified effect of NO_2_ on PM_10_.

#### 2.2.4. Model Testing and Sensitivity Analysis

Core models were tested based on white noise verification through calculating PACF of the residuals. With respect to the sensitivity analysis, we changed the df for long-term trend in interaction models, so as to examine the robustness of joint effect graphs and the ERRs (%) under stratification.

*p* ≤ 0.05 was considered statistically significant. All analyses were performed in R 3.3.1 [20] using the “rgl” and “mgcv” packages.

## 3. Results

Mortality, air pollution and meteorological data of the study area from 2006 to 2010 are described in Table 1. A total of 59,609 non-accidental deaths can be seen, with an average of 33 per day. Among them, cardiovascular and cerebrovascular diseases-related deaths accounted for 33.42% and 11.99% of the total non-accidental mortality, respectively. During the study period, the average daily concentrations of air pollutants were 71.79 μg/m^3^ for PM_10_ and 60.31 μg/m^3^ for NO_2_, both of which were higher than the standards of 70.00 μg/m^3^ for PM_10_ and 40.00 μg/m^3^ for NO_2_ in China [21].

**Figure 2:**
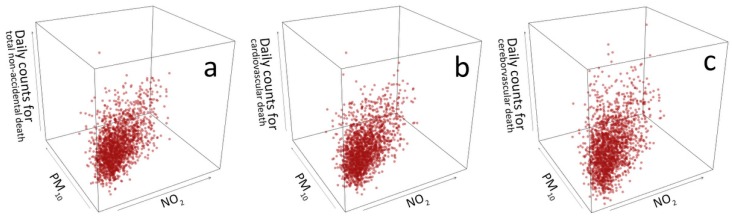
Three-dimension scatter plots of daily PM_10_, NO_2_ and mortality (2006–2010). This figure displays the correlation between daily PM_10_ concentrations, daily NO_2_ concentrations and daily non-accidental death counts in a three-dimensional way. The subplots a, b and c in this figure are the scatter plots between air pollution concentrations and three subsets of mortality (non-accidental death, cardiovascular death and cerebrovascular death), respectively.

Table 2 demonstrates that PM_10_ was positively correlated with NO_2_ (Pearson’s correlation coefficient of 0.75). Meanwhile, air pollutants were poorly correlated with each of the meteorological factors, with the coefficients of less than 0.20. Moreover, three-dimension scatter plots were drawn to simultaneously observe the correlations among PM_10_, NO_2_ and mortality. As shown in Figure 2, the dots mainly congregate around the diagonals of the graphs, along with similar uptrend for all three death causes, suggesting positive correlation among the two pollutants and mortality in Guangzhou.

In single pollutant models, the lag effects of PM_10_ and NO_2_ on mortality mainly occurred within the single lag of 2 days. Meanwhile, the greatest ERR% for each death cause was almost at the lag of moving average of 0–2 days (Table 3). Therefore, the effects of lag 0–2 days were chosen for both PM_10_ and NO_2_ for the further interaction analysis.

**Figure 3:**
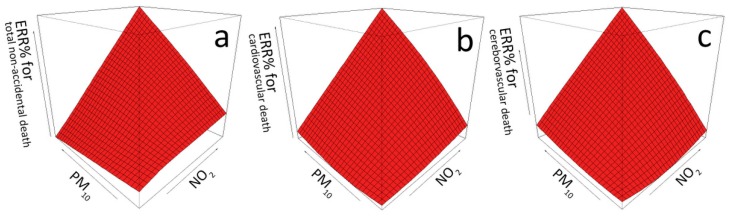
Joint effect graphs of lag 0–2 days PM_10_ and NO_2_ on mortality. This figure visualizes the strength change of excess relative risk percentage (ERR%) of PM_10_ and NO_2_ concentrations at lag of 0–2 days on mortality. Subgraphs a, b and c of the figure are joint effect graphs of the two air pollutants for three subsets of mortality (non-accidental death, cardiovascular death and cerebrovascular death), respectively.

The joint effect graphs of PM_10_ and NO_2_ at lag of 0–2 days are displayed in Figure 3. As can be observed, PM_10_ tends to be more lethal for all the three death causes accompanied by the increase in NO_2_ concentration. Furthermore, the slopes indicating ERRs (%) of NO_2_ have also become steeper with the increase in PM_10_. Moreover, risk surfaces in all the three subgraphs show a steady uptrend, indicating that the joint action of PM_10_ and NO_2_ might induce a greater effect on total non-accidental death, cardiovascular death and cerebrovascular death. However, the smoothing variable representing the concentrations of both PM_10_ and NO_2_ showed no statistically significant difference in the cerebrovascular death model.

Fitting the stratified models, effect of PM_10_ was found to be enhanced by the escalating level of NO_2_ at lag of 0–2 days. Notably, the greatest impact occurred in the presence of high NO_2_ levels (>76.14 μg/m^3^); otherwise, the risk of PM_10_ showed no statistical significance. The largest ERRs (%) of PM_10_ for total non-accidental death, cardiovascular death and cerebrovascular death were 0.46 (95% CI: 0.13, 0.79), 0.61 (95% CI: 0.06, 1.16) and 0.99 (95% CI: 0.17, 1.83), respectively (Table 4). Likewise, PM_10_ served as a positive modifier of NO_2_ on mortality in Guangzhou. NO_2_ demonstrated its strongest effects on total non-accidental death (ERR%: 0.92, 95% CI: 0.42–1.42) and cardiovascular death (ERR%: 1.20, 95% CI: 0.38–2.03) while PM_10_ was above the 75th percentile (>89.82 μg/m^3^). However, the risk on cerebrovascular mortality was lack of statistical significance (Table 5). In summary, results of the stratified analyses strengthen the evidence of interaction between PM_10_ and NO_2_ on non-accidental mortality, especially the cardiovascular diseases-related deaths.

In the white noise test, none of the absolute values of PACF were over 0.10 up to lag 12, except for the value for total non-accidental death model at lag 2, indicating the high fitting degrees of the core models. Besides, both the joint effects and the modified effects revealed no significant changes with the varying df for long-term trend, which had also verified the robustness of the results (Figure S1, Tables S1 and S2).

## 4. Discussion

The potential interaction between PM_10_ and NO_2_ and its effect on non-accidental mortality in Guangzhou was explored in the current study, to help better understand the health impacts of ambient air pollution in China. Existing epidemiological studies have generally investigated the interactions among air pollutants through assessing the mutual modified effects [5,6,9,12,22]. To some extent, stratification can reduce the collinearity between pollutants in the multi-pollutant model [23]. But stratified results alone may be inadequate to illustrate such relationships. Some researchers have attempted to estimate the interactive effects using other approaches [13,24]. Nevertheless, accepted method to tackle this issue is still lacking. In the present study, the bivariate model was introduced, which is suitable for qualitatively investigating the interaction between two continuous variables [13,25,26]. Afterwards, we further examined such interaction quantitatively using stratification analysis technique.

At lag of 0–2 days, we found greater interactive effect between PM_10_ and NO_2_ on mortality, which suggests that the risk of mortality would remarkably increase as the two air pollutants are both at high levels. This finding implies the necessity to issue health advice on time to communicate the health risk in the public when these two air pollutants raise simultaneously. Katsouyanni et al. [6] in APHEA2 study and Samoli et al. [5] in NMMAPS study also found that increased NO_2_ concentration was associated with greater PM_10_ effects on mortality. Additionally, other researchers have observed positive interactions between PM_10_ and NO_2_ on emergency hospital admission and cardiovascular mortality [7,13]. Combined with our results, this suggests that patients with chronic diseases, such as cardiovascular diseases, may be more vulnerable to the hazard of interaction between PM_10_ and NO_2_. However, an effect of such interactions on cerebrovascular mortality has not been found in the current study, which is inconsistent with the findings from Hong [19]. It is reasonable that there are various patterns of interactions among different populations and areas, which can be attributed to the intricacy of source contribution of air pollution, and the complex composition of particulate matter. For instance, the modified effect of NO_2_ on PM_10_ in the United States is less pronounced than that in Europe, which may be probably ascribed to the distinction of source contributions between cities in Europe and the United States [5,6]. Therefore, the conclusions should be interpreted with caution on this issue. We should consider the specific effect of interaction between PM_10_ and NO_2_ in each area, respectively. More importantly, the results should be cautiously extrapolated to other populations. Meanwhile, a consistent approach is also of vital significance to compare the results among different studies.

The interaction mechanism between PM_10_ and NO_2_ on human health remains unclear. In certain chemical processes, NO_2_ can transform into nitrate, which is one of the critical components of particulate matter. On the one hand, increased NO_2_ concentration will raise the nitrate proportion in particles, consequently resulting in greater health hazard of PM_10_. On the other hand, as a carrier of irritant gases, PM_10_ has the ability to absorb and transport NO_2_ into the human body [27], which will render a greater level of exposure. In addition, chemical reactions between NO_2_ and PM_10_ components occurring during the above processes may also be the underlying mechanism of the interaction [4,28]. Existing results have suggested that traffic-generated particles are more toxic, and likely to demonstrate a more remarkable interaction with NO_2_ [5,6]. In the meantime, vehicle emissions are becoming the predominant source of ambient air pollution in Guangzhou. It is possible that these two pollutants will induce a stronger interactive effect on mortality.

However, the current study is inevitably associated with several limitations. As a time series analysis, we mainly focused on the variation trends of variables, thereby inevitably ignoring the information at an individual level. The consequent ecology fallacy may reduce the accuracy of our results to some extent. Although we have explored the interaction both visually and quantitatively with consistent results in two models, however, the method adopted in the present study cannot completely distinguish the effect of NO_2_ from PM_10_, and vice versa, making it insufficient to draw the conclusion of synergy or antagonism. Meanwhile, the cutoffs of stratified analyses were selected with reference to previous studies [6,8], but whether such stratification reflects the actual interactive mode between PM_10_ and NO_2_ remains uncertain; ways of division are also greatly diverse in existing studies [13,19,23]; therefore, a more adequate approach is still urgently needed in this field. In addition, other pollutants have not been taken into account in the current study owing to the collinearity, but air pollutants present as a complex mixture in the ambient environment, and such pollutants may play an exaggerating or masking role in the effects of interaction between PM_10_ and NO_2_. Besides, the fine particles (PM_2.5_) and ultrafine particles (PM_0.1_) may cause even more intense adverse health effects than PM_10_, and their interactive relationships with other air pollutants have yet to be investigated. Therefore, it is necessary to assess the complete picture of interactions among various air pollutants on public health in the future. According to previous study, effects of PM_10_ and NO_2_ are stronger on the elderly population [16]. Likewise, the sensitivity to interaction between PM_10_ and NO_2_ may be different among different age groups. It is essential to identify the susceptible population of interactions among air pollutants, so as to subsequently develop the prevention strategy.

## 5. Conclusions

In conclusion, the current study contribute to better understanding the health effect of ambient PM_10_ and NO_2_ in China. Our results suggest the positive interactive effect between these two air pollutants on non-accidental mortality in Guangzhou. This finding may imply that the interactive effects of air pollutants should be taken into account when developing risk communication tools.

## Figures and Tables

**Table 1:** Distribution of daily mortality, air pollution concentrations and meteorological factors in Yuexiu and Liwan districts, Guangzhou, China (2006–2010).

Variables	Mean	SD	Percentiles				
			Min.	*P*_25_	*P*_50_	*P*_75_	Max.
Daily death counts							
Total non-accidental	32.66	7.88	11.00	27.00	32.00	37.00	81.00
Cardiovascular	10.92	4.24	1.00	8.00	10.00	13.00	36.00
Cerebrovascular	3.92	2.16	0.00	2.00	4.00	5.00	13.00
Air pollution concentrations (μg/m^3^)							
PM_10_	71.79	40.17	8.33	43.58	63.00	91.33	307.50
NO_2_	60.31	29.63	12.96	38.40	53.87	76.17	199.40
Meteorological factors							
Mean temperature (°C)	22.89	6.19	5.40	18.60	24.40	27.80	33.50
Relative humidity (%)	71.14	13.04	25.00	64.00	72.00	81.00	99.00

**Table 2:** Pearson’s correlations between daily air pollution concentrations and meteorological factors (2006–2010).

Variables	PM_10_	NO_2_	Mean Temperature	Relative Humidity
PM_10_	1.00	0.75	−0.16	−0.16
NO_2_		1.00	−0.15	−0.07
Mean temperature			1.00	0.17
Relative humidity				1.00

All coefficients are statistically significant.

**Table 3:** ERRs (%) with 95% confidence intervals for mortality for each 10 μg/m^3^ increment in PM_10_ and NO_2_ concentrations at different lag time.

Death Causes	ERR% (95% CI) of PM_10_	ERR% (95% CI) of NO_2_
Total non-accidental		
Lag 0	**0.31 (0.05, 0.57)** *	**0.67 (0.30, 1.04)** *
Lag 1	**0.40 (0.14, 0.66)**	**0.84 (0.46, 1.21)** *
Lag 2	**0.25 (0.01, 0.50)** *	**0.43 (0.07, 0.80)** *
Lag 3	−0.03 (−0.28, 0.22)	−0.01 (−0.36, 0.35)
Lag 0–1	**0.47 (0.18, 0.77)** *	**0.95 (0.53, 1.37)** *
Lag 0–2	**0.52 (0.20, 0.84)** *	**0.98 (0.53, 1.44)** *
Lag 0–3	**0.43 (0.08, 0.77)** *	**0.83 (0.34, 1.32)** *
Cardiovascular		
Lag 0	0.27 (−0.16, 0.70)	**0.67 (0.06, 1.28)** *
Lag 1	**0.49 (0.06, 0.92)** *	**0.93 (0.31, 1.56)** *
Lag 2	**0.54 (0.12, 0.96)** *	**0.80 (0.19, 1.41)** *
Lag 3	0.06 (−0.35, 0.47)	0.06 (−0.53, 0.66)
Lag 0–1	**0.51 (0.02, 1.00)** *	**1.01 (0.32, 1.70)** *
Lag 0–2	**0.70 (0.17, 1.24)** *	**1.21 (0.46, 1.97)** *
Lag 0–3	**0.64 (0.06, 1.21)** *	**1.07 (0.27, 1.88)** *
Cerebrovascular		
Lag 0	0.24 (−0.42, 0.91)	0.47 (−0.45, 1.40)
Lag 1	0.56 (−0.09, 1.22)	0.94 (−0.01, 1.89)
Lag 2	**1.14 (0.50, 1.77)** *	**1.21 (0.29, 2.15)** *
Lag 3	0.08 (−0.54, 0.71)	0.02 (−0.88, 0.93)
Lag 0–1	0.54 (−0.20, 1.29)	0.89 (−0.14, 1.93)
Lag 0–2	**1.02 (0.22, 1.83)** *	**1.28 (0.16, 2.41)** *
Lag 0–3	**0.89 (0.04, 1.75)** *	1.08 (−0.12, 2.28)

* The statistically significant effects are in bold.

**Table 4:** ERRs (%) with 95% confidence intervals for mortality for each 10 μg/m^3^ increment of lag 0–2 days PM_10_ across NO_2_ levels.

NO_2_ Level	Number of Days	ERR% (95% CI) of Lag 0−2 Days PM_10_		
		Total Non-Accidental Death	Cardiovascular Death	Cerebrovascular Death
Low	455	−0.16 (−0.90, 0.58)	−0.32 (−1.56, 0.94)	0.67 (−1.26, 2.63)
Medium	911	0.02 (−0.43, 0.47)	0.16 (−0.60, 0.92)	0.75 (−0.40, 1.92)
High	457	**0.46 (0.13, 0.79)** *	**0.61 (0.06, 1.16)** *	**0.99 (0.17, 1.83)** *

* The statistically significant effects are in bold. Cut-off points of NO_2_ level are the 25th and 75th percentiles of lag 0–2 concentration (39.90 and 76.14 μg/m^3^).

**Table 5:** ERRs (%) with 95% confidence intervals for mortality for each 10 μg/m^3^ increment of lag 0–2 days NO_2_ across PM_10_ levels.

PM_10_ Level	Number of Days	ERR% (95% CI) of Lag 0–2 Days NO_2_		
		Total Non-Accidental Death	Cardiovascular Death	Cerebrovascular Death
Low	456	0.70 (−0.33, 1.74)	1.20 (−0.52, 2.96)	0.13 (−2.47, 2.80)
Medium	910	**0.87 (0.18, 1.57)** *	0.98 (−0.17, 2.15)	0.38 (−1.34, 2.13)
High	457	**0.92 (0.42, 1.42)** *	**1.20 (0.38, 2.03)** *	1.04 (−0.18, 2.28)

* The statistically significant effects are in bold. Cut-off points of PM_10_ level are the 25th and 75th percentiles of lag 0–2 concentration (47.04 and 89.82 μg/m^3^).

## Supplementary Materials

The following are available online at www.mdpi.com/1660-4601/14/11/1381/s1, Figure S1: Joint effect graphs of lag 0–2 days PM_10_ and NO_2_ on mortality with different df of long-term trend, Table S1: ERRs (%) with 95% confidence intervals for mortality for each 10 μg/m^3^ increment of lag 0–2 days PM_10_ across NO_2_ levels with different dfs of long-term trend, Table S2: ERRs (%) with 95% confidence intervals for mortality for each 10 μg/m^3^ increment of lag 0–2 days NO_2_ across PM_10_ levels with different dfs of long-term trend.
