# Supplementary material for: The Interaction between Ambient PM10 and NO2 on Mortality in Guangzhou, China
Source: Int J Environ Res Public Health. 2017 Nov 13;14(11):1381. doi: 10.3390/ijerph14111381 (PMC5708020; doi:10.3390/ijerph14111381)
Supplement: Supplementary file 1 [file ijerph-14-01381-s001.pdf]

Article

The interaction between ambient PM_{10} and NO_2 on mortality in Guangzhou, China

Yuzhou Gu [1], Hualiang Lin [2], Tao Liu [2], Jianpeng Xiao [2], Weilin Zeng [2], Zhihao Li [2], Xiaojuan Lv [2] and Wenjun Ma [2,*]

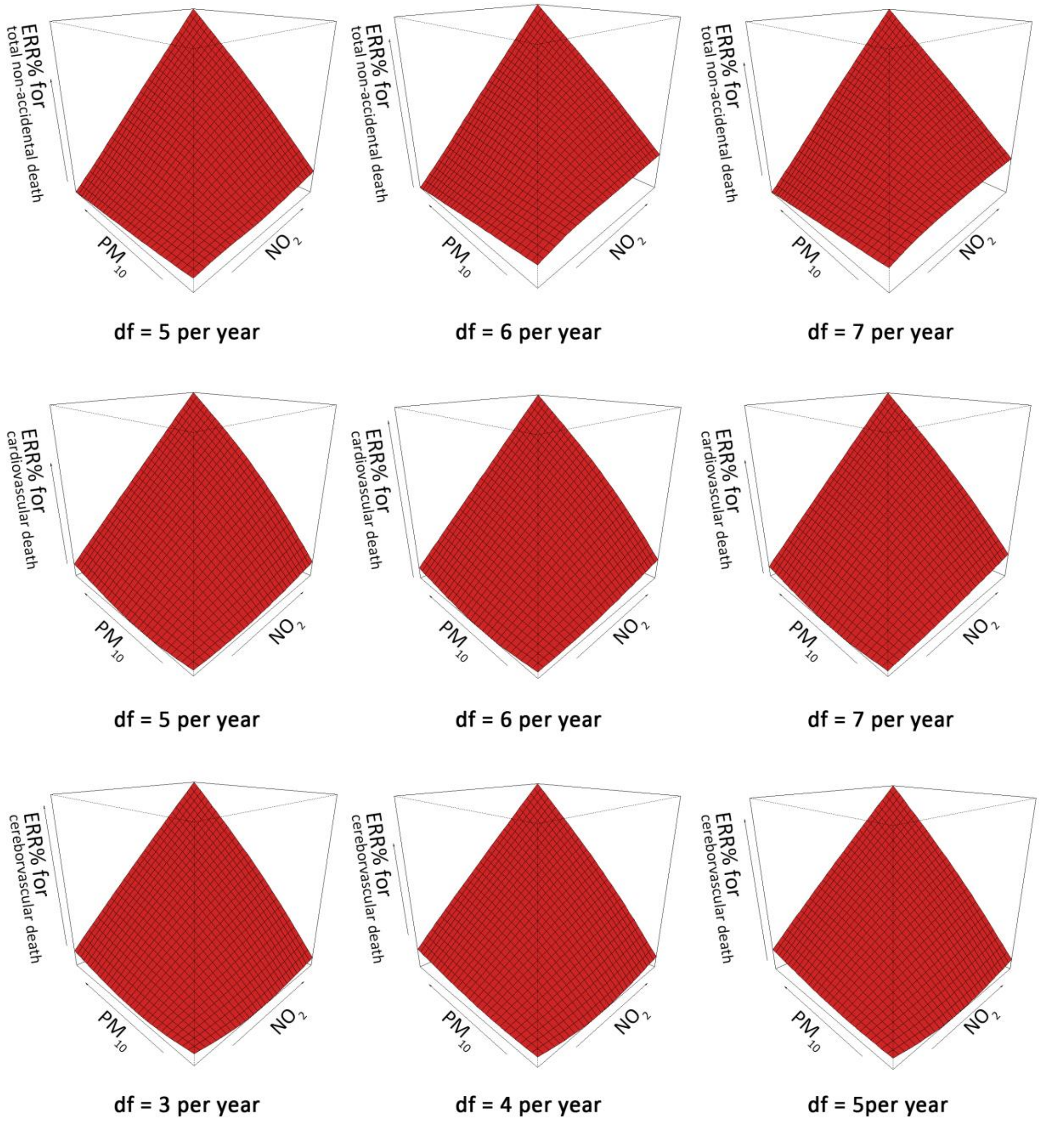

Figure S1. Joint effect graphs of lag0-2 days PM_{10} and NO_2 on mortality with different df of long-term trend.

This figure displays the joint effect graphs of lag0-2 days PM_{10} and NO_2 on mortality with different df of long-term trend in bivariate model. Subgraphs in three rows of the figure are joint effect graphs of the two air pollutants for three subsets of mortality (non-accidental death, cardiovascular death and cerebrovascular death) respectively.

Table S1. ERRs (%) with 95% confidence intervals for mortality for each 10 μg/m³ increment of lag0-2 days PM_{10} across NO_2 levels with different dfs of long-term trend.

**NO_2 level**	**Total non-accidental death**			**Cardiovascular death**			**Cerebrovascular death**		
	**Df of long-term trend (df/year)**			**Df of long-term trend (df/year)**			**Df of long-term trend (df/year)**		
	**5**	**6**	**7**	**5**	**6**	**7**	**3**	**4**	**5**
Low	0.04 (-0.69, 0.78)	-0.16 (-0.90, 0.58)	-0.20 (-0.94, 0.54)	-0.17 (1.40, 1.08)	-0.16 (-0.90, 0.58)	-0.46 (-1.70, 0.80)	0.31 (-1.56, 2.21)	-0.16 (-0.90, 0.58)	0.73 (-1.18, 2.68)
Medium	0.09 (-0.36, 0.54)	0.02 (-0.43, 0.47)	0.01 (-0.44, 0.47)	0.24 (-0.51, 1.00)	0.16 (-0.60, 0.92)	0.16 (-0.59, 0.93)	0.56 (-0.59, 1.72)	0.75 (-0.40, 1.92)	0.82 (-0.34, 1.99)
High	**0.52 (0.19, 0.85)**	**0.46 (0.13, 0.79)**	**0.41 (0.08, 0.74)**	**0.66 (0.12, 1.20)**	**0.61 (0.06, 1.16)**	0.55 (-0.01, 1.10)	**0.89 (0.07, 1.72)**	**0.99 (0.17, 1.83)**	**1.01 (0.18, 1.85)**

The statistically significant effects are in bold. Cut-off points of NO_2 level are the 25th and 75th percentiles of lag0-2 concentration (39.90 and 76.14 μg/m³).

Table S2. ERRs (%) with 95% confidence intervals for mortality for each 10 μg/m³ increment of lag0-2 days NO_2 across PM_{10} levels with different dfs of long-term trend.

PM_{10} level	Total non-accidental death			Cardiovascular death			Cerebrovascular death		
	Df of long-term trend (df/year)			Df of long-term trend (df/year)			Df of long-term trend (df/year)		
	5	6	7	5	6	7	3	4	5
Low	0.35 (-0.67, 1.37)	0.70 (-0.33, 1.74)	0.56 (-0.47, 1.61)	0.92 (-0.77, 2.64)	1.20 (-0.52, 2.96)	1.21 (-0.53, 2.98)	0.47 (-2.08, 3.09)	0.13 (-2.47, 2.80)	0.01 (-2.57, 2.66)
Medium	0.60 (-0.08, 1.27)	**0.87 (0.18, 1.57)**	**0.80 (0.10, 1.49)**	0.74 (-0.39, 1.88)	0.98 (-0.17, 2.15)	1.05 (-0.11, 2.23)	0.38 (-1.31, 2.10)	0.38 (-1.34, 2.13)	0.27 (-1.44, 2.01)
High	**0.81 (0.32, 1.30)**	**0.92 (0.42, 1.42)**	**0.81 (0.31, 1.31)**	**1.14 (0.33, 1.95)**	**1.20 (0.38, 2.03)**	**1.16 (0.33, 1.99)**	1.09 (-0.12, 2.32)	1.04 (-0.18, 2.28)	0.99 (-0.24, 2.23)

The statistically significant effects are in bold. Cut-off points of PM_{10} level are the 25th and 75th percentiles of lag0-2 concentration (47.04 and 89.82 μg/m³).
